# Primary health care research in COVID-19: analysis of the protocols reviewed by the ethics committee of IDIAPJGol, Catalonia

**DOI:** 10.1186/s12875-023-02025-5

**Published:** 2023-04-06

**Authors:** Anna Moleras-Serra, Rosa Morros-Pedros, Mónica Monteagudo, Cristina Vedia-Urgell, Ainhoa Gómez-Lumbreras

**Affiliations:** 1 Fundació Institut Universitari Per a La Recerca a L’Atenció Primària de Salut Jordi Gol I Gurina (IDIAPJGol), Barcelona, Spain; 2 Universitat Autònoma de Barcelona, Bellaterra (Cerdanyola del Vallès), Barcelona, Spain; 3 Direcció d’Atenció Primària Metropolitana Nord, Institut Català de La Salut, Barcelona, Spain; 4 College of Pharmacy, Department of Pharmacotherapy, University of Utah, Salt Lake City, USA

**Keywords:** COVID-19, Primary health care, Research, Ethics committees, Workload

## Abstract

**Background:**

Since March of 2020, the scientific community has been engaged a marathon to answer the different questions that COVID-19 pandemic has brought. During this time, Ethics Committees played an important role in reviewing the research protocols, COVID-19 or not, ensuring that the quality of scientific research is not relaxed by the hasty need for answers.

**Methods:**

Descriptive study from January 2019 to December 2021, comparing COVID-19 protocols to those not COVID-19 related protocols and comparing the work overload.

Variables related to the characteristics of the research protocols (i.e. study design, funding…), the principal investigators (gender, PhD degree, professional role…) and outcomes of the Ethics Committee process (requirements of modifications and time until approval) were analyze.

**Results:**

The number of sessions increased during COVID-19 pandemics (12 in 2019, 25 in 2020 and 18 in 2021). In total 751 protocols were evaluated during the study period; 513 (68.3%) had an observational design and 434 (57.8%) had no funding. The principal investigator was a woman in 491 (65.4%) studies and a General Practitioner in 330 (43.9%). The mean of the days until the protocol approval was 42.09 days (SD 60.2) with a decrease of 20.1 days from 2019 to 2021. A total of 614 (81.7%) protocols were approved, 336 (54.7%) within the first month after their initial evaluation. Less than half of the protocols were COVID-19 related (208, 44.3%). The COVID-19 protocols main topics were impact on the population (71, 34.1%); and COVID-19 pharmacological treatments (including vaccines) showed a higher increase in 2021 (37, 30.3%).

**Conclusions:**

Despite the work overload during the pandemic due to the increase in the number of meetings and protocols, the IDIAPJGol EC reviewed all of them (COVID-19 or not) adapting to the new situation but according to its criteria of good practices to provide a quick response in the EC opinion. In Primary Health Care the most study designs have been observational studies, many of them with no funding and led by GPs. In 2021 there was an increase in the number of protocols focused on drugs, most likely related to COVID-19 vaccines studies.

## Introduction

In December 2019 a novel coronavirus causing a severe acute respiratory syndrome with high mortality rates was identified as SARS-CoV2 in Wuhan (China); the world was hit by the disease COVID-19, which was declared as global pandemic by the World Health Organization (WHO) on 11 March 2020 [1]. In Spain, it was declared on 14 March 2020 [2].

Since the first COVID-19 outbreak in March 2020 the scientific community has been engaging a marathon to answer the different questions that the pandemic has brought. Conduction of clinical trials to get the most efficacy treatments, observational studies to characterize the clinical and epidemiological features and vaccines development to provide immunity have been one of the main aims of health institutions to achieve quickly results. By December 2021 over 7,353 studies on COVID-19 have been registered in clinicaltrials.gov [3]. In Spain, the Spanish Medicines Agency (AEMPS) has registered 207 studies to that same date [4].

COVID-19 pandemic has forced health systems update their assistance and research protocols periodically and the Different medical societies have published guidelines to keep on with the clinical assistance during the pandemic Regulatory bodies such as European Medicine Agency (EMA), Food and Drug Administration (FDA) or Spanish Agency of Medicines and Medical Devices (AEMPS) have also adapted research to the pandemic including guidance for researchers to ensure trials of medicinal products are safe and ethical [5–9].

In this mobilization of health and research teams, the Ethics Committees (EC) or Institutional Review Boards (IRB) have played and play an important role in reviewing of all COVID-19 protocols ensuring that the protocol quality and ethics quality of scientific research is not relaxed by the hasty need for answers. In March 2020, WHO published that pandemics such as COVID-19 do not overrule the need to uphold ethical standards [10]. The challenge to EC/IRB during the COVID-19 era is how to maintain research standards of quality and integrity and to ensure the protection of human beings, all in record times, as results are needed as soon as possible. The European Network of Research Ethics Committees published a position paper about the responsibility of research EC during the COVID-19 pandemic focused on the prioritization of protocols related to COVID-19, the need for updated processes for ethics reviews and the maintained importance of informed consent of participants in COVID-19 related research [11]. The Spanish Bioethics Committee published a report in December 2020 with specific ethical and legal requirements for research in the framework of the COVID-19 pandemic [12]. Spain has a universal coverage health system funded mainly from citizens taxes which allows provision of healthcare free of charge with partial coverage for outpatient prescribed medications [13] The Spanish health care system went through a decentralization in the late 1900 and Catalunya created the “Catsalut” (Catalan Health Service) in charge of providing public healthcare for Catalonia [14]. The main healthcare provider for Primary Health Care (PHC) in Catalonia is the Catalan Health Institute (ICS) [15]. PHC is the access for patients to the healthcare system. The principal settings are primary care centers which primary care teams are formed by primary care physicians, nurses, pediatricians, dentistries and social workers [16]. The Spanish PHC has become the first contact with health services for people with mild COVID-19 symptoms, as well as the institution to answer doubts and control people in quarantine from their homes [17]. PHC has been leading and helping the collapsed health system during the pandemic without leaving aside its mission not only to investigate but also to provide tools to improve the health and well-being of citizens [18]. The main research potential of PHC is population-based clinical studies, but the research on PHC it is not exempt from difficulties such as lack of time, pressure on healthcare assistance, scarce resources, or lack of recognition, especially in comparison with the major research in other fields closer to the hospital settings like oncology or cardiology [19]. In Spain, there are few ECs of PHC institutions, among them the IDIAPJGol EC, which evaluates research protocols from the public health system of the Catalonian PHC setting. The IDIAPJGol EC was created on August 1996. It was the first EC to be accredited for PHC in Spain. The ICS considered the EC a key element to promote research in PHC, where the diagnosis of the more prevalent diseases takes place and where most medicines are prescribed. The IDIAPJGol EC supervises the 87% of PHC centers of Catalonia, including the ICS, which is the main health provider in Catalonia with more than 400 primary care centers [20].

The aim of this research is to describe the characteristics of the research protocols and their principal investigators reviewed by the IDIAPJGol EC and to compare the workload of the committee during the COVID-19 pandemic in Catalonia, Spain to a year before the starts of the pandemic.

## Methods

This is an observational retrospective descriptive and comparative study from the protocols evaluated by the IDIAPJGol EC from January 2019 to December 2021, differentiating COVID-19 protocols.

The IDIAPJGol EC uses its own software of management system called Comprehensive Management of Research [Gestió Integral de la Recerca (GIR) in Catalan], https://portal.idiapjgol.org:6443/gir/login/index.php?entorn=IDIAP) which collects all the variables related to researchers and research protocols conducted in ICS PHC centers and other health institutions providers of Health Department of Catalonia.

The following GIR variables were used for the study analysis:

- Research protocol variables: study design (interventional, observational, qualitative study, and others), funding source (external or internal grant (grants from IDIAPJGol or ICS), private funding, no funding), COVID-19 study objective topic (epidemiology, pharmacotherapy, diagnosis and seroprevalence, and population impact).
- Principal Investigator (PI) characteristics: gender, professional category, PhD degree, membership to a research group.
- Outcomes of the EC process: result of the evaluation (approval, modification required, denied) and time until approval.

A sub-analysis has been conducted comparing COVID-19 versus non-COVID-19 protocols evaluated since the pandemic hit (April 2020 – December 2021).

### Statistical analysis

Annual descriptive statistics were used to summarize the different variables. Continuous variables were reported as mean and standard deviation (SD) as appropriate, categorical variables were reported as proportions (%). For the days to approval variable, these were stratified in 5 categories (0 days, 1–31 days, 32–90 days, 91–180 days and more than 180 days). Continuous variables were compared using the T-test or Mann–Whitney U test and categorical variables were compared using the χ2 or Fisher exact test when applicable.

The results were described using hazard ratios with a 95% confidence interval (CI 95%) and value *p* < 0.05 was considered significant. All statistical analyses were performed using the statistical software package Statistical Package for Social Science (SPSS) version 25.0.

## Results

**Fig. 1:**
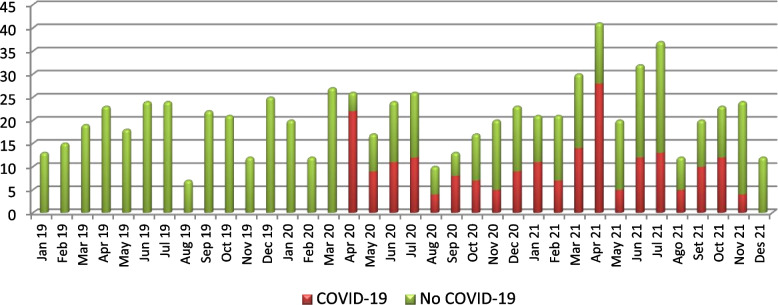
Number of Protocols reviewed per month by Ethics Committee during the study period Red: COVID-19 Green: non COVID-19

Since the beginning of the COVID-19 pandemic the number of protocols reviewed by the IDIAPJGol EC has increased, as well as the number of sessions. With fluctuations the number of protocols evaluated monthly raised from 2019 to 2021, with 2 peaks in April 2021 and July 2021 (41 and 37 protocols, respectively); Focused on COVID-19 protocols, the peak was in April 2020 and in April 2021. (See Fig. 1 for the monthly distribution).

In total 751 protocols were evaluated during the study period, 223 (29.7%) in 2019, 235 (31.3%) in 2020 and 293 (39.0%) in 2021 (Table 1). A total of 513 research protocols (68.3%), had an observational design and more than half (434, 57.8%) had no funding. The PI was a women in 491 (65.4%) protocols; a GP in 330 (43.9%) protocols; 272 of the PIs (36.2%) hold a PhD degree, and 203 (27.0%) are members of an accredited research group.

**Table 1:** Descriptive of the Ethics Committee Activity by Year

	**2019**	**2020**	**2021**	**TOTAL**
**N (%)**	**223 (29.7)**	**235 (31.3)**	**293 (39.0)**	**751 (100.0)**
**Study design**				
Interventional study	42 (18.8)	43 (18.3)	39 (13.3)	124 (16.5)
Observational study	155 (69.5)	161 (68.5)	197 (67.2)	513 (68.3)
Qualitative study	18 (8.1)	18 (7.7)	40 (13.7)	76 (10.1)
Others^a^	8 (3.6)	13 (5.5)	17 (5.8)	38 (5.1)
**Funding**				
External/Public grant	26 (11.7)	39 (16.6)	52 (17.7)	117 (15.6)
Internal grant	39 (17.5)	14 (6.0)	9 (3.1)	62 (8.3)
Private funding	36 (16.1)	34 (14.5)	68 (23.2)	138 (18.4)
No funding	122 (54.7)	148 (63)	164 (56)	434 (57.8)
**PI characteristics**				
Gender (Female)	156 (70.0)	152 (64.7)	183 (62.5)	491 (65.4)
Holds a PhD degree (yes)	78 (34.5)	90 (38.3)	104 (35.5)	272 (36.2)
Member of a research group (yes)	56 (24.8)	71 (30.2)	76 (25.9)	203 (27)
**Professionals**				
General Practitioners (GP)	104 (46.6)	106 (45.1)	120 (41.0)	330 (43.9)
Physicians other than GP	27 (12.1)	34 (14.5)	36 (12.3)	97 (12.9)
Pediatricians	7 (3.1)	10 (4.3)	4 (1.4)	21 (2.8)
Physicians (Gynecologist)	2 (0.9)	2 (0.9)	4 (1.4)	8 (1.1)
Nurse	49 (22.0)	47 (20.0)	63 (21.5)	159 (21.2)
Other health professionals	18 (8.1)	10 (4.3)	14 (4.8)	42 (5.6)
Pharmacists	8 (3.6)	7 (3.0)	11 (3.8)	26 (3.5)
Biostatisticians/IT	0 (0.0)	0 (0.0)	5 (1.7)	5 (0.7)
Others	3 (1.3)	3 (1.3)	11 (3.8)	17 (2.3)
Unknown	5 (2.2)	16 (6.8)	25 (8.5)	46 (6.1)
**Number of sessions held**	12	25	18	55
**EC Results**				
Approval	185 (83.0)	188 (80.0)	241 (82.3)	614 (81.6)
Days until approval				
0	47 (25.4)	55 (29.3)	66 (27.4)	168 (27.4)
1–30	41 (22.2)	54 (28.7)	73 (30.3)	168 (27.4)
31–90	51 (27.6)	42 (22.3)	75 (31.1)	168 (27.4)
91–180	30 (16.2)	28 (14.9)	25 (10.4)	83 (13.5)
> 6 months	16 (8.6)	9 (4.8)	2 (0.8)	27 (4.4)
Modification required	38 (17.0)	44 (18.7)	51 (17.4)	133 (17.8)
Withdrawn	0 (0.0)	3 (1.3)	0 (0.0)	3 (0.4)
Denied	0 (0.0)	0 (0.0)	1 (0.3)	1 (0.1)
**Days to be approved (mean, SD)**	55 (73.0)	41.5 (65.4)	34.9 (40.6)	42.9 (60.2)
**Covid-19 study objective topic**		86 (41.3)	122 (58.7)	208 (100.0)
Epidemiology		26 (30.2)	24 (19.7)	50 (24.0)
Pharmacological treatment		9 (10.5)	37 (30.3)	46 (22.1)
Diagnoses tests		24 (27.9)	17 (13.9)	41 (19.7)
Population impact of COVID-19		27 (31.4)	44 (36.1)	71 (34.1)

^a^ Others: economist, sociologists, engineer, journalists, geography and history, trade and marketing

The mean number of days for a protocol to be approved by the IDIAPJGol EC in the overall study period was 42.09 days (SD 60.2), 2019 to 2021 it decreased in 20.1 days (from an average of 55.0 (SD 73.0) days in 2019 to 34.9 (SD 40.6) days in 2021). By the end of the study period 614 (81.6%) protocols were approved, 336 (54.8%) at least in the first 30 days, half of them at their initial submission and the rest after required modifications.

Analyzing the COVID-19 protocols by objective topic, most of them were related to COVID-19 impact on the population (71, 34.1%), followed by those protocols focus on pharmacological treatment (vaccines included) (46, 22.1%). These pharmacological ones showed the highest increase from 9 (10.5%) in 2020 to 37 (30.3%) in 2021 while a decrease in the epidemiology ones (from 26, 30.2% in 2020 to 24, 19.7% in 2021).

Table 2 shows the comparison between COVID-19 protocols and the rest of protocols during the period of pandemics (April 2020 to December 2021). Of all the protocols reviewed since the beginning of the COVID-19 pandemic (March 2020), 208 (44.3%) were COVID-19 protocols being 87 (41.8%) in 2020 and 121 (58.2%) in 2021.

**Table 2:** Comparative between COVID-19 and Non-COVID-19 Protocols (Apr 2020 – Dec 2021)

Total protocols 469	COVID-19	No COVID-19	*p* value
**N (%)**	**208 (44.3)**	**261 (55.7)**	**0.016**
**Study design**			
Interventional study	17 (8.2)	48 (18.4)	0.001
Observational study	162 (77.9)	161 (61.7)	0.000
Qualitative study	18 (8.7)	34 (13.0)	0.134
Others^a^	11 (5.3)	18 (6.9)	0.473
**Funding**			
External/Public grant	47 (22.6)	37 (14.2)	0.018
Internal grant	7 (3.4)	12 (4.6)	0.501
Private funding	33 (15.9)	64 (24.5)	0.021
No funding	121 (58.2)	148 (56.7)	0.749
**PI characteristics**			
Gender (Female)	122 (58.7)	174 (66.7)	0.002
Holds a PhD degree (yes)	86 (41.3)	88 (33.7)	0.019
Research group membership (yes)	74 (35.6)	61 (23.4)	0.004
**Professionals**			
General Practitioners (GP)	100 (48.1)	91 (34.9)	0.004
Physicians other than GP	34 (16.3)	33 (12.6)	0.255
Pediatricians	7 (3.4)	6 (2.3)	0.485
Physicians (Gynecologist)	0 (0.0)	6 (2.3)	0.008
Nurse	29 (13.9)	66 (25.3)	0.002
Other health professionals	10 (4.8)	14 (5.4)	0.786
Pharmacists	6 (2.9)	10 (3.8)	0.575
Biostatisticians/IT	2 (1.0)	3 (1.1)	0.844
Others	4 (1.9)	10 (3.8)	0.228
Unknown	16 (7.7)	22 (8.4)	0.771
**EC Results**			
Approval	175 (84.1)	209 (80.1	0.257
Days until approval			
0	46 (26.3)	57 (27.3)	0.828
1–30	16 (9.1)	54 (25.8)	0.009
31–90	67 (38.3)	65 (31.1)	0.197
91–180	44 (25.1)	28 (13.4)	0.192
> 6 months	2 (1.1)	5 (2.4)	0.362
Modification required	32 (15.4)	49 (18.8)	0.335
Withdrawn	1 (0.5)	2 (0.8)	0.696
Denied	0 (0.0)	1 (0.4)	0.279
**Days to be approved (mean, SD)**	33.3 (45.0)	40.1 (52.1)	0.101

^a^Others: Others: economist, sociologists, engineer, journalists, geography and history, trade and marketing

In relation to the study design, we found significant differences in the observational studies [162, (77.9%) in COVID-19 protocols vs. 161 (61.7%) in non-COVID-19 ones, *p* < 0.01] and in interventional studies [17 (8.2%) vs. 48 (18.4%), *p* < 0.01), COVID-19 and non-COVID-19, respectively]. Significant differences were also found for COVID-19 protocols funded by external or public grants [47 (22.6%) vs. 37 (14.2%), *p* = 0.018 for COVID-19 and non-COVID-19, respectively], as well as for those with private funding, [33 (15.9%) vs. 64 (24.5%), *p* = 0.021 for COVID-19 and non-COVID-19 protocols, respectively].

Comparing the PI COVID-19 protocols, fewer had a woman as a PI [122 (58.7%) vs. 174 (66.7%); *p* = 0.002]; and more COVID-19 protocols’ PI were GPs [100 (48.1%) vs. 91 (34.9%); *p* = 0.004)] and those non COVID-19 had more nurses as PI [29 (13.9%) vs. 66 (25.3%); *p* = 0.002].

COVID-19 protocols approved within the first month after the evaluation (1–31 days) were non-COVID-19 ones [67 (38.3%) vs. 54 (25.8%), *p* = 0.009].

## Discussion

The IDIAPJGol EC work overload has increased from 2019 during 2020 and this increase is maintained during 2021. This overload has been not only in the number of research protocols to be reviewed but also in the number of meetings held by the EC. Other ECs in Spain and abroad have published similar trends in their work overload [21–23].

The number of observational studies increased in the research carried out in PHC in Catalonia and one of the possible reason lies in the PHC research goals, the disease epidemiology whose approach could be describing patients’ characteristics, monitoring signs and symptoms and trying to identify preventive and predictive factors. Also, PHC can analyze the impact of the disease at a community level [19].

There was an increase in the number of protocols without funding in 2020 and 2021, we would like to think that at a time of urgent need for answers our health care workers standards prevailed, so many studies were conducted even with no funding. Research that even involved overexertion and extra work for PHC teams was carried out, making PHC workers invest their own time and resources in trying to get the desired answers. The great awareness of the Spanish researchers obtained a quick response from the government, which soon published external public calls [24].

However, in the COVID-19 protocols we could see an increase in private funded projects, especially in 2021, as well as an increase in drug studies possibly due to safety studies on COVID-19 vaccines, which are mainly conducted by the pharmaceutical industry [25]. In this context, PHC plays an important role due to its accessibility to reach the population, allowing the recruitment of individuals from the entire population [26].

The COVID-19 studies were mainly led by GPs due to their involvement in fight against the pandemic. Likewise it seems that COVID-19 studies were carried out by more experienced researchers since a higher percentage of PI hold a PhD and are members of an accredited research group.

In order to be able to maintain the rhythm of reviews and not delay the EC opinions the IDIAPJGol EC, followed the recommendations of EUREC [11], to adopt new strategies, such as increasing in the frequency of meetings from monthly to weekly and meeting virtually. This has been a common strategy adopted by other EC/IRB, reducing the time until the approval without delaying the assessment of all the protocols [27, 28]. With the procedural changes applied, especially the increase in the number of meetings, protocols were approved in 21 days less in 2021 respect to 2019. Moreover, online meetings have additional benefits such as saving time and costs, as well as avoiding unnecessary movements [29]. It has to be mention that the IDIAPJGol EC has never diminished its ethical requirements and has maintain all the strictness, COVID-19 pandemic included, according to the WHO ethic guidance in research during outbreaks and the Spanish Bioethics Committee COVID-19 report [10, 12].

COVID-19 protocols took an average of 7 days less to be approved than non-COVID-19 protocols favoring the progress of the COVID-19 research at a time of exceptional urgency. Although there is a reasonable doubt about COVID-19 protocols having been able to maintain the same research quality than the other protocols. Some articles have shown that research protocols assessed by EC during the pandemic were often incomplete and the urgency made that researchers did not consider all the aspects related to the research [30]. A systematic review of the research methodological quality during the pandemic showed that the clinical research was mainly observational with modest scores in methodological quality. COVID-19 papers were associated with a lower methodological quality score when published with a shorter time frame and in lower impact factor (IF) journals [31].

### Limitations and strengths

This study analyzed a PCH EC so no information on those studies conducted in the hospital setting has been considered. COVID-19 research done in PHC instead of hospitals may have differed in their objectives. The data have been extracted from a management database but is not error-free. Likewise, the urgency to give a quick opinion and the adaptation to the news ways of work during the lockdown may have led to missing information on the funding entity, total amount achieved or research grant.

## Conclusion

Despite of the work overload during the pandemic due to the increase in the number of protocols, the IDIAPJGol EC kept with its activity, mainly by incrementing the number of meetings. (COVID-19 or not) this led to a quick response in the EC opinion reducing considerably the time until approval.

## Data Availability

The datasets generated and analyzed during the current study are not publicly available due to these are in the institution management system and it is an administrative database used to the proper functioning of the institution and for its internal dashboard but they are available from the corresponding author on reasonable request.

## Abbreviations

AEMPS: Spanish Agency of Medicines and Medical Devices
EC: Ethics Committee
EMA: European Medicines Agency
EUREC: European Network of Research Ethics Committees
FDA: Food and Drug Administration
GIR: Comprehensive Management of Research
GP: General Practitioner
IDIAPJGol: Foundation University Institute for Primary Health Care Research Jordi Gol i Gurina (IDIAPJGol)
IRB: Institutional Review Board
NICE: National Institute for Health and Care Excellence
PHC: Primary Health Care
PI: Principal Investigator
SD: Standard Deviation
SPSS: Statistical Package for Social Science
WHO: World Health Organization
